# P-1740. Development of In-House Primers for Rapid Detection of Echinocandin Resistance in Candida glabrata

**DOI:** 10.1093/ofid/ofaf695.1911

**Published:** 2026-01-11

**Authors:** Madain S Alsanea, Abdulrahman M AlSweed, Fatimah AlHamlan, Ahmed Alqahtani, Reem AlMaghrabi

**Affiliations:** King Faisal Specialist Hospital & Research Center, Riyadh, Ar Riyad, Saudi Arabia; King Faisal Specialist Hospital & Research Center, Riyadh, Ar Riyad, Saudi Arabia; King Faisal Specialist Hospital & Research Center, Riyadh, Ar Riyad, Saudi Arabia; King Faisal Specialist Hospital & Research Center, Riyadh, Ar Riyad, Saudi Arabia; King Faisal Specialist Hospital and Research Center, Riyadh, Ar Riyad, Saudi Arabia

## Abstract

**Background:**

*Candida glabrata* is an opportunistic pathogen that has emerged as a major cause of invasive candidiasis, particularly among immunocompromised patients. The high mortality rates associated with *C. glabrata* infections, ranging from 40% to 60%, highlight the urgent need for rapid and accurate detection methods for antifungal resistance. The traditional methods face several limitations, including high cost and prolonged turnaround time (TAT). Therefore, this study aimed to design in-house primers to detect echinocandin resistance mutations rapidly and more accurately.

**Figure:**
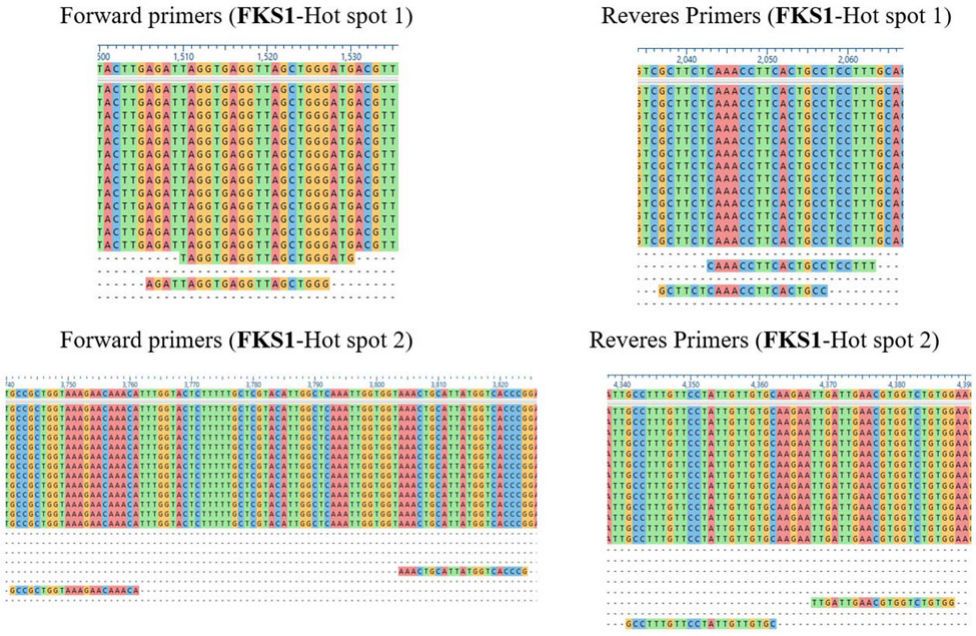
FKS1 primers targeting HotSpot1 and HotSpot 2 In-house designed primers aligned to representative sequences. The figure shows the binding sites of the primers.

**Figure:**
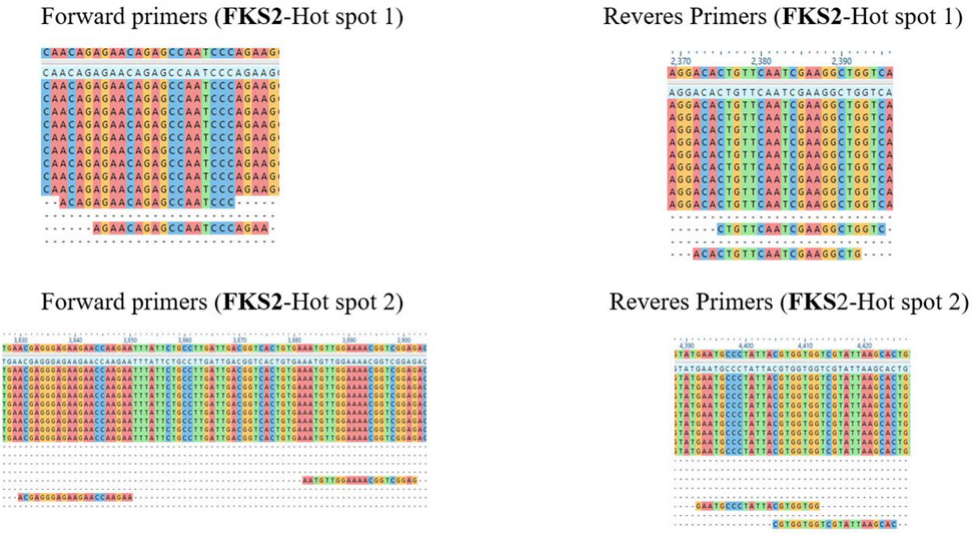
FKS2 primers targeting HotSpot1 and HotSpot 2 In-house designed primers alignment to representative sequences illustrates the conserved regions of the binding sites.

**Methods:**

The chromosomal locations of *FKS1* (chromosome G) and *FKS2* (chromosome K) were identified using the NCBI Gene database and visualized through the Candida Genome Database JBrowser tool. More than 20 published studies were reviewed to compile a list of resistance-associated mutations confined to the hotspot regions, which informed the primer design. BLASTn was utilized to obtain sequences for multiple sequence alignment, ensuring primer sites flanked conserved regions. Primers were designed using Primer-BLAST and assessed with OligoAnalyzer for optimal melting temperature, GC content, and specificity.

**Figure:**
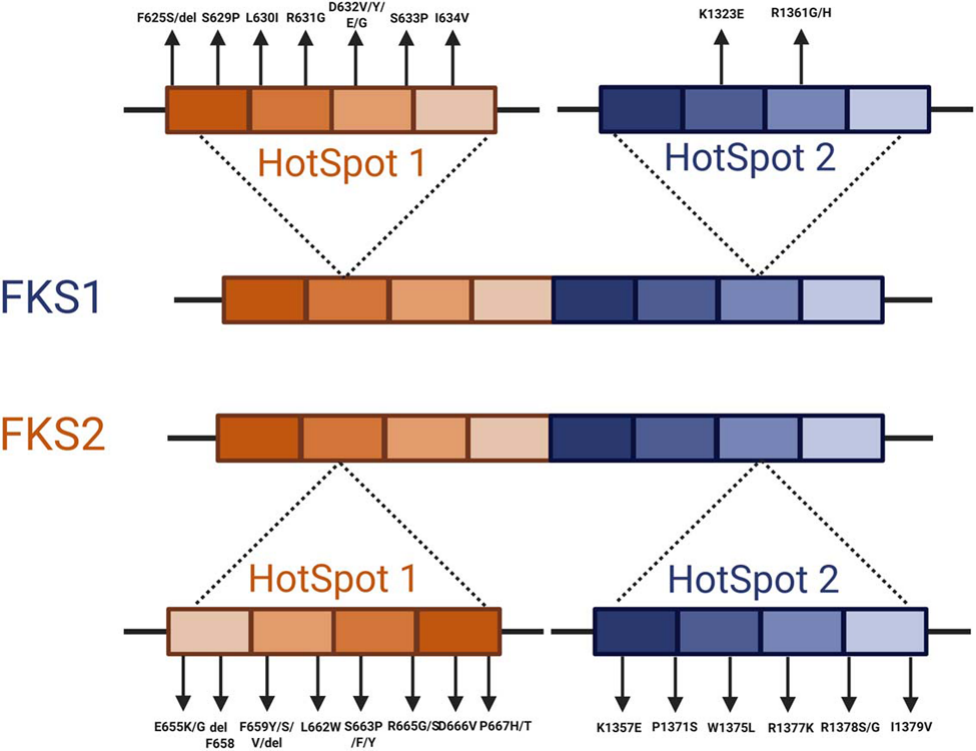
FKS1 and FKS2 hot spots mutations The illustration shows the reported mutation in the hot spot regions in FKS1 and FKS2, where the most mutations are detected.

**Figure:**
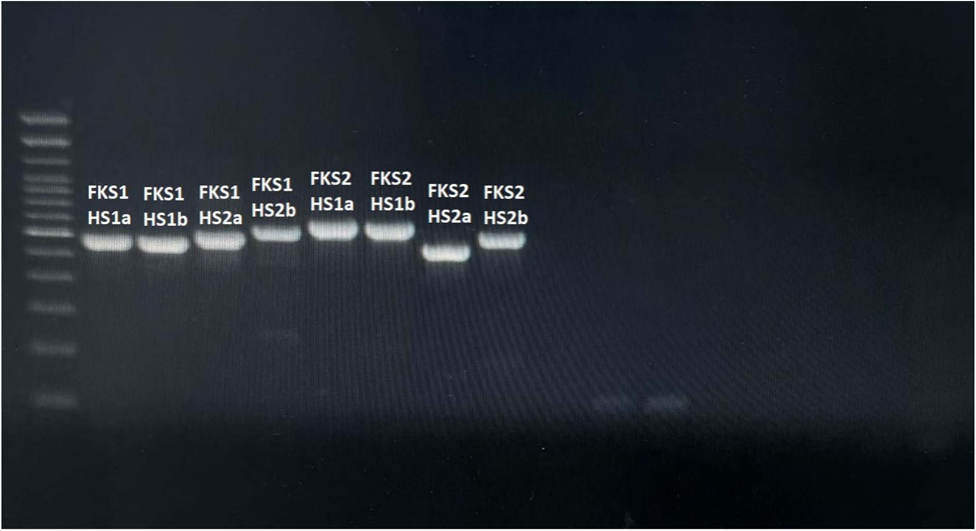
Gel electrophoresis results showing the successful amplification of the primers Following the in-silico tests, the primers were optimized and tested using patient samples. The figure demonstrates the amplicons of all designed primers. These amplicons were sequenced and used the BLAST tool to check the results, which showed a high level of specificity and sensitivity of the designed primers.

**Results:**

In-house primers were effectively designed to amplify hot spot 1 and hot spot 2 regions of the FKS1 and FKS2 genes in Candida glabrata. PCR amplification produced clear bands corresponding to the expected product sizes. Sequencing of these amplicons confirmed the specific amplification of the target regions when aligned with the reference genome. Although no novel mutations were identified in the current sample, the successfully amplified regions correspond precisely to the known echinocandin resistance-associated hotspot areas. Reported mutations within these regions, as described in previous literature, were compiled for reference to support future comparative analysis and resistance screening applications.

**Conclusion:**

The designed primers aim to support the rapid screening of clinical isolates for echinocandin resistance, enabling timely therapeutic decisions. This in-house assay could contribute to improved antifungal stewardship, early detection of resistant strains, and more effective management of candidiasis in at-risk patients.

**Disclosures:**

All Authors: No reported disclosures

